# Flower color polymorphism of a wild *Iris* on the Qinghai-Tibet plateau

**DOI:** 10.1186/s12870-023-04642-9

**Published:** 2023-12-09

**Authors:** Zhi-Li Zhou, Guang-Yan Wang, Xi-Long Wang, Xiao-Juan Huang, Zhang-Shichang Zhu, Lin-Lin Wang, Yong-Ping Yang, Yuan-Wen Duan

**Affiliations:** 1 Germplasm Bank of Wild Species, Yunnan Key Laboratory of Crop Wild Relatives Omics, Institute of Tibetan Plateau Research at Kunming, Kunming Institute of Botany, Chinese Academy of Sciences, Chinese Academy of Sciences, Kunming, Yunnan 650201 China; 2 Tibet Plateau Institute of Biology, Lhasa, Xizang 850001 China

**Keywords:** Anthocyanins, Pollen-to-ovule ratio, *F3H*, Expression, SNPs, Selection

## Abstract

**Background:**

Flower color plays a crucial role in attracting pollinators and facilitating environmental adaptation. Investigating the causes of flower color polymorphism and understanding their potential effects on both ecology and genetics can enhance our understanding of flower color polymorphism in wild plant.

**Results:**

In this study, we examined the differences of potential male and female fitness between purple- and yellow- flower individuals in *Iris potaninii* on the Qinghai-Tibet Plateau, and screened key genes and positively selective genes involved in flower color change. Our results showed that yellow flower exhibited a higher pollen-to-ovule ratio. Yellow flowers were derived from purple flowers due to the loss of anthocyanins, and *F3H* could be an essential gene affecting flower color variation though expression regulation and sequence polymorphism in this species. Furthermore, our findings suggest that genes positively selected in yellow-flowered *I. potaninii* might be involved in nucleotide excision repair and plant-pathogen interactions.

**Conclusions:**

These results suggest that *F3H* induces the flower color variation of *Iris potaninii*, and the subsequent ecological and additive positive selection on yellow flowers may further enhance plant adaptations to alpine environments.

**Supplementary Information:**

The online version contains supplementary material available at 10.1186/s12870-023-04642-9.

## Background

Flower color is an important signal that shapes plant interactions with pollinators and the surrounding environment [1]. Flower color is evolutionarily labile, and thus flower color change above species level is common in flowering plants [2–4]. Since pollinators would perceive flower color change, inter-specific variations of flower color could play a key role in reproductive isolation mediated by pollinator preference and pollinator shift, leading to the conclusion that genes involved in color change are considered to be speciation genes [3, 5]. In contrast, intra-specific flower color changes (flower color polymorphism) are generally unusual but not rare. For stable occurrence of flower color polymorphism within populations, flower color changes are tightly correlated with biotic [6], abiotic stress [7], or genetic variation [8], which are accompanied by frequency-dependent selection (including negative-frequency selection and over-dominant selection), balancing selection, or neutral processes [9]. The numeric proportion between male and female gametes is the result from the balances of increasing pollen dispersion and seed production in plant species [10], and the differences of plant fitness induced by flower color change may indicate the different pollinator functional groups and pollination efficiencies. Thus, pollen-ovule ratio is an important indicator of mating system [11] and pollination efficiency [12], both of which consist of the reproductive strategies of plant species with flower color polymorphism.

Flower color is determined by the accumulation of pigments (flavonoids, carotenoids, and betalains) in the petals. Flavonoids are polyphenolic metabolites, in which anthocyanins are the main compounds responsible for orange to blue coloration. Anthocyanins are synthesized in the flavonoid biosynthetic pathway and anthocyanin biosynthetic pathway. Genes underlying flower color change have been studied extensively, particularly in relation to color variations based on anthocyanins. For example, a frameshift mutation in *DFR* (*dihydroflavonol 4-reductase*, a flavonoid structural gene in the flavonoid biosynthetic pathway) of *Mimulus lewisii* leads to the absence of anthocyanin production in pink flowers, resulting in the emergence of white flowers [13]. However, anthocyanin biosynthesis varies among different plant species and color polymorphism scenarios. In *Mimulus*, while *NEGAN* is responsible for the transition to anthocyanin-pigmented petals in *M. luteus* var. *variegatus* [14], *cis*-regulatory change of *LAR1* (*leucoanthocyanidin reductase*) alters flower color by redirecting dihydroflavonol towards flavonol biosynthesis from the flavonoid biosynthetic pathway [4]. Besides, while flower color changes can easily induce pollinator mediated selection or plant-environment interaction, the genetic effects resulting from intra-specific flower color variation remain unclear.

**Fig. 1:**
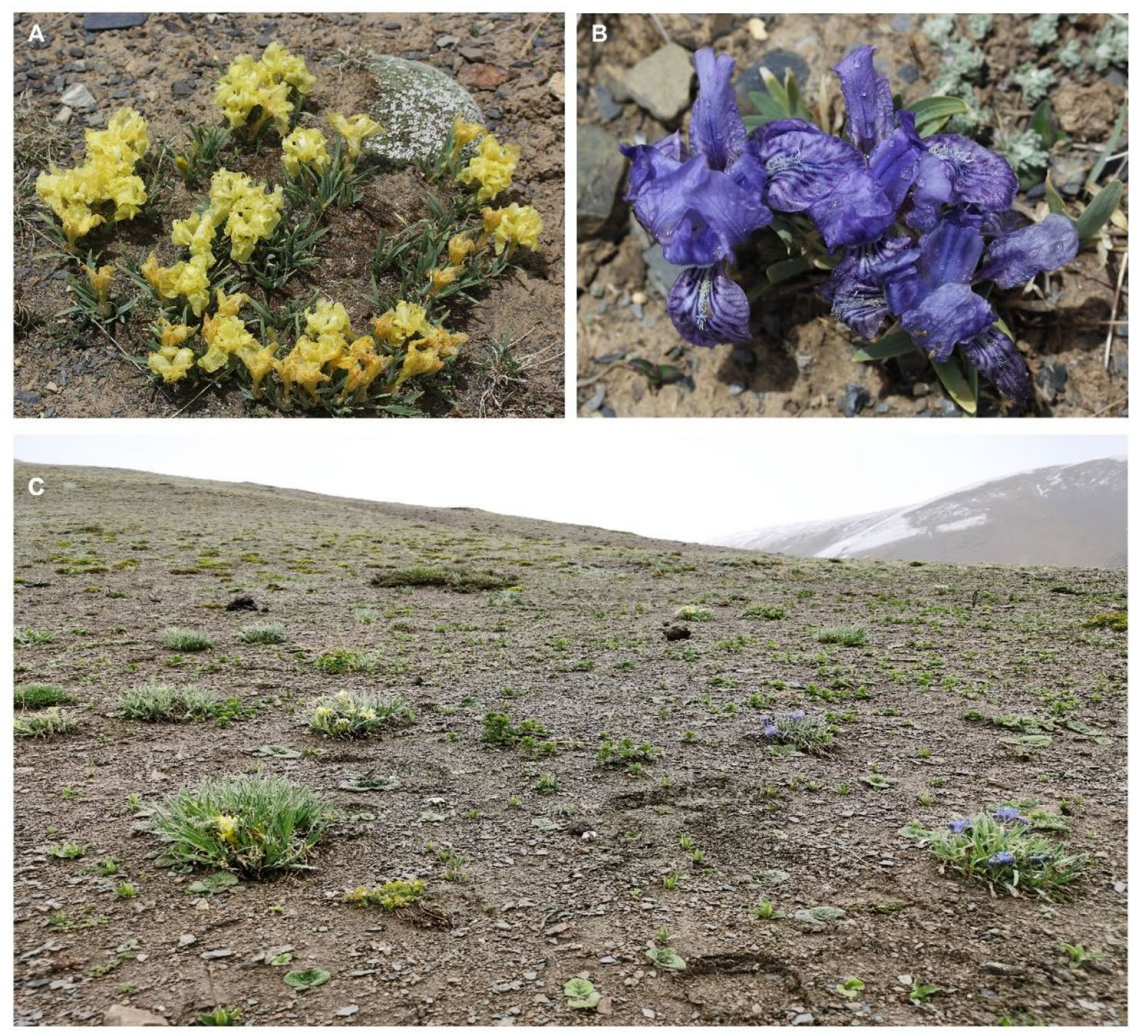
Yellow- flowered **(A)** and purple-flowered **(B)***I. potaninii* and their sympatric distribution **(C)**

The genus *Iris* L. comprises around 300 perennial species that are distributed in temperate regions across the Northern Hemisphere. In *Iris potaninii* Maxim., we found two flower-colored (yellow and purple, Fig. 1A, B) plants occurred thoroughly in sympatry at small geographical scales in field expeditions on the Qinghai-Tibet Plateau (Fig. 1C). In this study, we were motivated to investigate the differences of pollen-to-ovule between the yellow and purple morphs in sympatric population, with an aim to examine the potential effect of color variation on male and female fitness indicated by pollen-to-ovule ratio. Then, by combining anthocyanin measurements, differential gene expression, and selection analysis, we screened potential candidate genes involving flower color dimorphism and the genetic consequences accompanied by flower color variation in *I*. *potaninii*. This study could greatly contribute to our understanding of on the ecological effects and genetic mechanisms of intra-specific flower color variation in alpine plants.

## Results

### Differences in pollen-to-ovule (P/O) ratio and anthocyanins

**Fig. 2:**
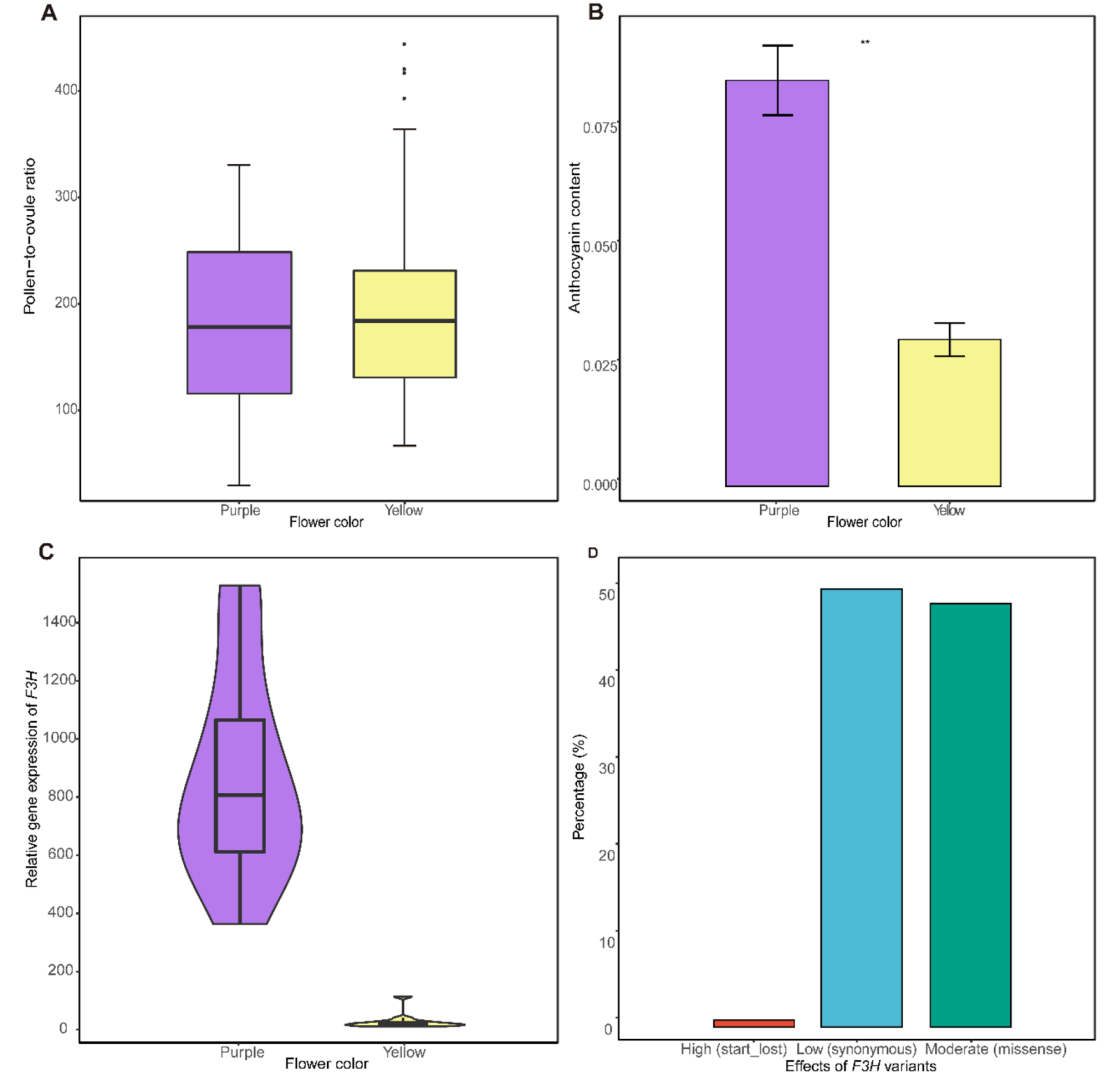
Ecological and genetic differences between purple-flowered and yellow-flowered *I. potaninii*. **(A)** Pollen-to-ovule ratio of yellow flowers is slightly higher than purple flowers. **(B)** The total contents of anthocyanins in purple flowers are significantly higher in comparison with yellow flowers (T-test, P = 0.001, asterisk indicates the 0.01 level). **(C)** Flavonoid structural gene *F3H* shows significantly higher expressional level in purple-flowered plants than yellow-flowered ones (logFC > 1, FDR < 0.05). **(D)***F3H* gene is consisted of one (0.84%) start_lost variant, 58 (48.74%) missense variants, and 60 (50.42%) synonymous variants. The start_lost variant and missense variants may deactivate or decline the gene activity of *F3H*, thus affect the synthesis of anthocyanins and the purple coloration of flowers

To investigate the impact of color variation on the fitness of male and female, we assessed the pollen and ovule production of the yellow and purple flowers. Generally, pollen number per flower was similar in both yellow and purple flowers (Figure S1A). However, purple flowers exhibited a significantly higher ovule production compared to yellow flowers (P = 0.01, Figure S1B), leading to a slight increase in pollen-to-ovule ratio of yellow flowers (Fig. 2A).

Since anthocyanins are responsible for orange to blue coloration, we compared the total content of anthocyanins and the main compound of anthocyanins between purple and yellow flowers. In flowers of the purple morph, the total contents of anthocyanins were significantly higher in comparison with yellow flowers (P < 0.01, Fig. 2B). Besides total content, the main compound of anthocyanins differed between purple and yellow flowers (Figure S2). Although both m/z 935 and m/z 919 were observed in the main peaks of purple (Figure S2A, B, D) and yellow morphs (Figure S2F, G, I), the MS^2^ fragment peaks at m/z 303 (delphinidin) were only present in compounds of m/z 935 and m/z 919 in purple flowers (Figure S2C, E, H, J). Therefore, delphinidin derivatives (m/z 935 and m/z 919) were the main compounds in purple flowers, whereas no delphinidin derivatives was identified as the dominant anthocyanins in yellow flowers.

### Transcriptome assembly and annotation of *I. Potaninii*

By transcriptome sequencing of 26 samples (13 individuals for each color-morph, Table S1), we *de novo* assembled 79,704 sequences from all color-morph individuals of *I. potaninii*, with N50 of 1,428 (Table 1). Integrity assessment revealed that a total of 1,332 (82.5%) complete BUSCOs (1,307 single-copy and 25 duplicated BUSCOs) annotated in the 1,614 BUSCOs groups, indicating that the assembly was of credible quality (Figure S3). For transcriptome annotation, 19,640 transcripts were assigned to the GO database, including 17,810 transcripts annotated with molecular function, 10,308 transcripts annotated with biological process, and 3,818 transcripts annotated with cellular component (Figure S4). KAAS analyses demonstrated that 9,521 transcripts associated with 410 KEGG pathways. Besides the largest annotated pathways of metabolic pathways and biosynthesis of secondary metabolites, we paid attention to genes associated with anthocyanin biosynthesis, which were responsible for anthocyanins-based color polymorphism. Altogether 96 genes were involved in the flavonoid biosynthetic pathway and anthocyanin biosynthetic pathway, including several flavonoid structural genes (*CHS*, *CHI*, *F3H*, *ANS*, *DFR*, and *FLS*, Table 2). Most of these flavonoid structural genes had considerable gene copy number, indicating the potential for functional redundant or functional differentiation of these copies.

**Table 1:** Basic information of transcriptome assemblies

Species	Transcript number	N50	BUSCO score (%)
*I. potaninii*	79,704	1,428	82.5
yellow-flowered *I. potaninii*	86,754	1,484	89.8
purple-flowered *I. potaninii*	86,142	1,475	92
*I. loczyi*	49,452	2,055	76.8
*I. atropurpurea*	41,522	1,757	91.6

**Table 2:** Information of genes involved in the flavonoid biosynthetic pathway and anthocyanin biosynthetic pathway

Gene	Description	KO	No.all^a^	No.up^b^	No.down^c^
*F3H*	Naringenin 3-dioxygenase [EC:1.14.11.9]	K00475	10	1	0
*CYP73A*	Trans-cinnamate 4-monooxygenase [EC:1.14.14.91]	K00487	2	0	0
*CCoAOMT*	Caffeoyl-CoA O-methyltransferase [EC:2.1.1.104]	K00588	13	0	0
*CHS*	Chalcone synthase [EC:2.3.1.74]	K00660	19	0	0
*CHI*	Chalcone isomerase [EC:5.5.1.6]	K01859	4	0	0
*ANS*	Anthocyanidin synthase [EC:1.14.20.4]	K05277	2	0	0
*FLS*	Flavonol synthase [EC:1.14.20.6]	K05278	1	0	0
*CYP98A*	5-O-(4-coumaroyl)-D-quinate 3’-monooxygenase [EC:1.14.14.96]	K09754	2	0	0
*UGT75C1*	Anthocyanidin 3-O-glucoside 5-O-glucosyltransferase [EC:2.4.1.298]	K12338	3	0	0
*BZ1*	Anthocyanidin 3-O-glucosyltransferase [EC:2.4.1.115]	K12930	5	0	0
*LAR*	Leucoanthocyanidin reductase [EC:1.17.1.3]	K13081	1	0	0
*DFR*	Dihydroflavonol 4-reductase/flavanone 4-reductase [EC:1.1.1.219 1.1.1.234]	K13082	4	0	0
*PGT1*	Phlorizin synthase [EC:2.4.1.357]	K22845	3	0	0
*HCT*	Shikimate O-hydroxycinnamoyltransferase [EC:2.3.1.133]	K13065	26	0	0

^a^ Total gene number.

^b^Number of upregulated genes in purple-flowered samples.

^c^Number of downregulated genes in purple-flowered samples.

*We obtained appropriate copyright permission to use the KEGG results depicted in Table 2

### Differentially expressed genes between purple and yellow flowers

To unveil the genetic mechanisms of the color variations, we conducted differential expression analysis with the assembly of two color-morph individuals of *I. potaninii* as reference. Altogether nine genes showed differential expression (logFC = 1, P < 0.05) between purple-flowered and yellow-flowered samples of *I. potaninii* (Table 3, Figure S5). Of all the differentially expressed genes (DEGs), one flavonoid structural gene *F3H* participating in the flavonoid biosynthetic pathway showed significantly higher expressional level in purple-flowered plants than yellow-flowered ones (Fig. 2C; Table 3), which was in accordance with our qRT-PCR result (Figure S6) and the color variation in *I. potaninii* (Fig. 2B). In contrast, the other structural genes involved in the flavonoid biosynthetic pathway and anthocyanin biosynthetic pathway (Tables 2 and 3) showed similar expression levels in both yellow-flowered and purple-flowered samples (Figure S7, Table 3), and other nine gene copies of *F3H* also did not express differently between the two-colored flowers. All these results suggested the vital role of *F3H* gene and this *F3H* gene copy in determining the anthocyanin content and color variation of *I. potaninii*.

**Table 3:** Differentially expressed genes between yellow-flowered and purple-flowered samples

Gene	Description	logFC	logCPM	PValue	FDR
TRINITY_DN2545_c11_g1	Flavanone 3-hydroxylase (F3H)	5.010972	8.824025	5.86E-37	2.54E-32
TRINITY_DN10825_c1_g1	Chemosensory protein 4 (CSP4)	6.116867	-0.97727	2.18E-10	4.74E-06
TRINITY_DN254435_c0_g1	Odorant-binding protein 6 (Obp6)	5.88171	-1.13555	3.65E-09	5.28E-05
TRINITY_DN20665_c0_g3	/	5.869861	0.203943	1.87E-07	0.002024
TRINITY_DN64671_c0_g1	/	-7.37173	-0.01215	4.13E-07	0.003581
TRINITY_DN12997_c2_g1	Membrane steroid-binding protein 1	-5.49745	3.669161	1.65E-06	0.011954
TRINITY_DN56294_c1_g1	/	-7.51858	0.117561	4.96E-06	0.030755
TRINITY_DN34461_c0_g2	/	-6.7548	-0.50296	7.92E-06	0.042223
TRINITY_DN9434_c0_g1	Peptidyl-tRNA hydrolase 2 isoform X2	4.32578	-0.97929	8.76E-06	0.042223

### SNPs genotyping of *F3H* genes

For SNPs genotyping, 4,846,194 polymorphic sites were generated by aligning all the color-morph individuals against the reference transcriptome of *I. potaninii*. After filtering with read depth, missing rate, and heterozygosity, 1,378,683 sites were retained. Since *F3H* genes were indicated to determine the color variation of *I. potaninii*, we further targeted the SNPs distributions of ten *F3H* gene copies in purple-flowered and yellow-flowered individuals. Among the ten gene copies of *F3H*, only the differentially expressed *F3H* gene contained 119 variants between two color-morphs samples. By annotating these variants, we found that the *F3H* gene consisted of one (0.84%) start_lost variant, 58 (48.74%) missense variants, and 60 (50.42%) synonymous variants (Fig. 2D). Of the 13 yellow-flowered individuals, the start_lost variant in 5 individuals caused start codon to be mutated into a non-start codon, which may lead to the loss of function of *F3H*. Besides, several missense variants occurred in yellow morph might change the color effectiveness of F3H protein. Thus, by sequence polymorphism and expression regulation, gene activity of the *F3H* deactivated or declined, which may highly affect the synthesis of anthocyanins and thus the purple coloration of flowers.

### Single copy genes screening

**Fig. 3:**
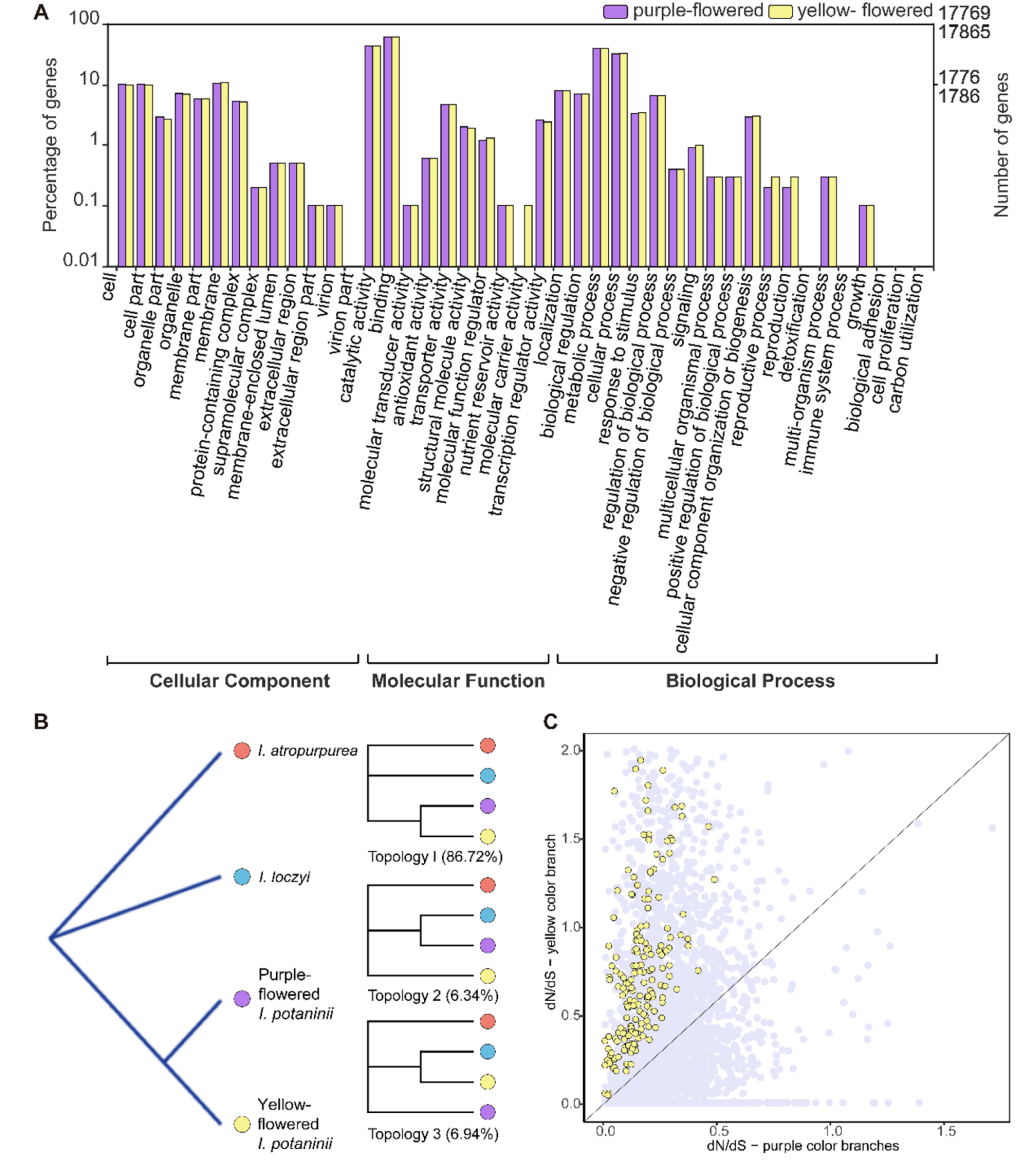
Additive genetic effects triggered by flower color variation of *I. potaninii*. **(A)** Annotated number differences occur in Gene Ontology between two color morphs of *I. potaninii*. **(B)** Phylogenetic analyses from 6,589 strict single-copy orthologs groups. Most single copy orthologs support yellow-flowered *I. potaninii* as sister to purple-flowered *I. potaninii* (Topology 1, 86.72%). **(C)** Protein evolution of 6,589 single copy orthologs among purple-flowered *I. potaninii*, yellow-flowered *I. potaninii*, *I. loczyi*, and *I. atropurpurea.* Differentially evolving genes with higher ω_yellow_ than ω_purple_ are indicated by yellow points

Besides the assembly of *I. potaninii*, we obtained the transcriptome assemblies of purple-flowered *I. potaninii*, yellow-flowered *I. potaninii*, and two purple-flowered outgroups (*I. loczyi* and *I. atropurpurea*). All these assembled sequences obtained high N50 values and BUSCO assessed scores, suggesting these data were of high quality for subsequent analyses (Table 1, Figure S3). For transcriptome annotation, annotated number differences occurred in Gene Ontology between two color morphs of *I. potaninii* (Fig. 3A). Specifically, a total of 29 transcripts were annotated in yellow-flowered *I. potaninii* with pollen-pistil interaction function (biological process category, classification of reproductive process), while only 26 transcripts involved in the process of pollen-pistil interaction for purple-flowered *I. potaninii*.

We further identified 6,589 strict single-copy orthologs groups (1:1:1:1) among purple-flowered *I. potaninii*, yellow-flowered *I. potaninii*, and two purple-flowered outgroups (*I. loczyi* and *I. atropurpurea*) and used them in phylogenetic analyses. Phylogeny illustrated a sister relationship between purple-flowered *I. potaninii* and yellow-flowered *I. potaninii*. Besides, 5,705 groups (86.72%) of orthologs also supported yellow-flowered *I. potaninii* as sister to purple-flowered *I. potaninii* (Topology 1; Fig. 3B), whereas limited phylogenies indicated a sister relationship between purple-flowered *I. potaninii* and *I. loczyi* or between yellow-flowered *I. potaninii* and *I. loczyi* (6.34%, Topology 2 or Topology 3; Fig. 3B). Since the most recent common ancestor of *Iris* has been putatively identified as purple flowers [15], We indicated that yellow flowers are derived from purple flowers of *I. potaninii*.

### Selection in yellow-flowered *I. Potaninii*

Branch model analysis was performed for differentially evolving events occurred in foreground branch (yellow-flowered *I. potaninii*). Among the 6,589 strict single-copy orthologs groups, 444 orthologs (6.74%) differentially evolved between yellow-flowered and purple-flowered species. Specifically, a total of 313 differentially evolving genes (70.50%) showed significantly higher ω_yellow_ than ω_purple_ (Fig. 3C). Branch-site model tests were then performed for signs of positive selection contributed to the elevated ω in yellow-flowered branch. Altogether 160 genes were under positive selection in foreground branch (yellow-flowered *I. potaninii*). GO and KEGG annotation of these positively selective genes detected several biological processes associated with stress, especially for nucleotide excision repair and plant-pathogen interaction (Table S2, Table 4).

**Table 4:** Positively selective genes associated with stress in yellow-flowered I. potaninii

Gene	Description	KEGG/GO annotation
IY|DN44065_c1_g1_i1	RNASEH2A; ribonuclease H2 subunit A	DNA replication
IY|DN2922_c0_g1_i11	RFA2; replication factor A2	DNA replication/ Nucleotide excision repair/ Mismatch repair
IY|DN51770_c1_g1_i1	RFC3_5; replication factor C subunit 3/5	Nucleotide excision repair/ Mismatch repair
IY|DN3543_c0_g1_i20	DDB1; DNA damage-binding protein 1	Nucleotide excision repair
IY|DN2205_c0_g1_i7	RAD23; UV excision repair protein RAD23	Nucleotide excision repai
IY|DN2561_c0_g1_i2	MEKK1; mitogen-activated protein kinase kinase kinase	Plant-pathogen interaction

*We obtained appropriate copyright permission to use the KEGG results depicted in Table 4

## Discussion

Flower color polymorphisms are common across the *Iris* species, and the most recent common ancestor of *Iris* has been putatively identified as purple flowers [15]. In our study, both phylogeny of four species and most single-copy orthologs show that yellow-flowered *I. potaninii* as sister to purple-flowered *I. potaninii* and the clustering of yellow-flowered *I. potaninii* and purple-flowered *I. potaninii* to two purple-flowered outgroups (*I. loczyi* and *I. atropurpurea*) **(**Fig. 3B**)**. Based on previous observations that yellow-flowered plants could be derived from purple species in *Iris*, our results suggest that yellow-flowered *I. potaninii* mainly derive from purple-flowered *I. potaninii*.

Changes of flower color may be associated with pollinator shift [16–19], but our field observation suggest that both purple-flowered plants and yellow-flowered plants recruit bumblebees as pollinators. For outcrossing plant species with early flowering phenology, pollen limitation of seed production is generally dominant since pollinator abundance could be low [20, 21]. Therefore, plant species with early flowering phenology might increase resource allocation to pollinator attraction. In this context, yellow flowers of *I. potaninii* might be more attractive to bumblebees than purple ones, which could be mirrored by following evidences. Compared with the limited reduction of pollen number in yellow flowers than purple flowers, lower ovule production of yellow flowers could result from the reduced resource allocation to female fitness that might be re-allocated to flower attraction (Fig. 2A). The resulting elevation of pollen-to-ovule ratio might indicate that pollinators could be more effective on yellow flowers. However, all these speculations could only be demonstrated with future field observations. Additionally, in comparison with purple flowers, more expressed transcripts of yellow-flowered plants are involved in the process of pollen-pistil interaction than purple-flowered plants, suggesting the sufficient pollen recognized genes and high efficiency of pollen utilization in yellow flowers.

Most *Iris* plants with pink, purple, or red flowers are anthocyanin-based [15]. In our study, the total contents of anthocyanins were significantly higher and delphinidin derivatives were the main compounds in purple flowers whereas little anthocyanins occurred and no delphinidin derivatives was identified as the dominant anthocyanins in yellow flowers. Mutations of flavonoid structural genes in the flavonoid biosynthetic pathway were reported to underlie the loss of anthocyanins. In the subspecies of Scarlet Gilia (*Ipomopsis aregata*), the lower expression and SNP variants of *DFR* gene drives flower color shift from red to white [22]. In our study, between the two-color morphs of *I. potaninii*, the relative expression level of *F3H* gene in purple flowers was significantly higher than that in yellow flowers (Fig. 2C, Figure S6, Table 3). Furthermore, start_lost variant and missense variants were included in the coding region of *F3H* for yellow-flowered *I. potaninii* (Fig. 2D), indicating coding sequence variation in anthocyanin biosynthesis pathway could disturb genes function of anthocyanin biosynthetic pathway and generate flower color difference. As a key biosynthetic enzyme in the anthocyanin pathway, *F3H* is directly associated with anthocyanin contents and flower color appearance. Therefore, both expressional and genetic evidences suggest that *F3H* could be a key gene affecting flower color variation in *I. potaninii*.

The occurrence of color polymorphism within populations may be caused by stabilizing selection driven by abiotic and biotic agents or by an absence of selective disadvantage on color [9]. Besides the ential increase of attraction to bumblebees, yellow flower color can strengthen the adaptation of *I. potaninii* to the abiotic and biotic environment at high altitudes (Table 4). For example, of the positively selective genes in yellow-flowered *I. potaninii*, *RFA2* regulates nucleotide excision repair and mismatch repair process [23, 24], while *MEKK1* is mainly involved in the plant-pathogen interaction [25, 26]. These positively selective genes associated with DNA repair and pathogen resistance might further contribute to the enhanced adaptations to alpine environments of yellow-flowered *I. potaninii*.

## Conclusions

In this study, our results suggest that yellow flowers might be more attractive to bumblebees than purple ones owing to the elevated ratio of pollen-to-ovule. Yellow flowers are derived from purple flowers of *I. potaninii* due to the loss of anthocyanins, and *F3H* may be a key gene inducing flower color polymorphism in this species. The positively selective genes in yellow-flowered *I. potaninii* might further enhance plant adaptations to alpine environments. Our comprehensive studies on *I. potaninii* could be an important case to witness the consequences of the flower color variation.

## Methods

### Plant material

*Iris potaninii* Maxim. is a perennial herb growing in hillsides at high elevation (> 3000 m) of Gansu province, Qinghai province, and Xizang of China. *Iris potaninii* begins to flower from middle May, and could be one of plant species with earliest flowering phenology on the Qinghai-Tibet Plateau. Flowers of *I. potaninii* are purple (Fig. 1A) and yellow (Fig. 1B), and the two groups of plants with different flower color are identified to occur widely after examinations of specimens. In the field, the two-colored plants are generally sympatric and mixed thoroughly. Our preliminary observations suggested that bumblebees were frequent visitors to both purple and yellow flowers, although visitation rates of bumblebees were low to the two-colored flowers. Two populations of *I. potaninii* around Nakchu, Xizang were selected for ecological studies and sample collection, where both purple and yellow flowers are abundant and mixed thoroughly (Table S1, Fig. 1C). The annual mean temperature was − 3.55 °C and − 3.12 °C, and the annual precipitation was 676 mm and 589 mm, for the two populations respectively. For transcriptome sequencing, flower buds of *I*. *potaninii* were collected from 26 plants in two populations (one flower bud for each plant), including 13 purple individuals and 13 yellow individuals (nine purple-flowered plants and nine yellow-flowered plants from Chali population, four purple-flowered plants and four yellow-flowered plants from Dirl population). Besides, the purple-flowered species of *I. loczyi* Kanitz and *I. atropurpurea* Baker were chosen as outgroups. Flower buds of *I. loczyi* were collected from Xizang and Qinghai province (Table S1), while transcriptome sequencing data of *I. atropurpurea* were downloaded from a previous study [27]. Information on all samples were presented in Table S1. For anthocyanin measurement, four opening purple flowers and four opening yellow flowers were collected from Chali population.

All plant materials were collected by Zhi-Li Zhou and Yuan-Wen Duan, and were identified by Prof. Yuan-Wen Duan (one of the corresponding authors in Kunming Institute of Botany, Chinese Academy of Sciences, China). The voucher specimens (Table S1) were deposited at the herbarium of Kunming Institute of Botany, Chinese Academy of Sciences, China (voucher number of purple-flowered *I. potaninii*: IP-JL-001(from Chali) and IP-DR-003 (from Dirl); voucher number of yellow-flowered *I. potaninii*: IP-JL-002 (from Chali) and IP-DR-004 (from Dirl); voucher number of *I. loczyi*: IL-YPC-001 (from Yangpachen) and IL-XNC-002 (from Xiaonanchuan). The ecological studies and sample collections of *I. potanini* and *I. loczyi* were permitted by the local government. The use of plant flowers in this study complied with all local, national or international guidelines and legislation concerning research involving plants.

### Differences in pollen-to-ovule (P/O) ratio

To estimate pollen and ovule production of the yellow and purple flowers, ten purple-flowered plants and 25 yellow-flowered plants were selected. One flower bud from each plant was carefully collected and fixed separately in FAA solution (formalin: acetic acid: ethanol at a ratio of 5:5:90 by volume). The anthers of each bud were dissected from the stamens, and pollen grains were dispersed in 5 ml micro-centrifuge tubes containing 2 ml FAA solution. For each sample, pollen number was counted using a microscope in ten replicates of 10 µl and then total pollen production of each flower was calculated. Ovule number of each flower was counted using a stereoscope. The pollen-to-ovule (P/O) ratio of each flower was calculated as dividing total number of pollen grains per flower by the total number of ovules. Independent T-test was employed to compare the differences between purple and yellow flowers.

### Anthocyanin measurement

To examine total anthocyanin contents and the anthocyanin classifications of purple and yellow flowers, we collected four fresh flowers of each color morph and kept them in liquid nitrogen. In laboratory, petals were squashed and the resultant powders were dissolved in 20% MeOH solution and sonicated at 20℃ for 20 min. After centrifugation (6,000 g for 20 min), the residue was then re-extracted with MeOH for additional three times. The extraction was diluted by a fourth volume of water without concentration. Total anthocyanin contents were quantified by UV-visible spectra (530 nm) with the standard compound of delphinidin-3-O-rhamnoside chloride.

Ultra-high performance liquid chromatography (HPLC) system equipped with an Agilent ZORBAX SB-C18 column (150 mm × 4.6 mm, 5 μm, Agilent Technologies Inc., America) was employed to examine the anthocyanin types with the gradient of solvent A (0.1% formic acid in water) and solvent B (acetonitrile). The flow rate was set up to 0.8 ml min^− 1^ and 10 µl of each flower morph was injected, with the column temperature of 60 ℃. The ESI (electrospray ionization) source was conducted in positive mode for anthocyanins with the monitored wavelength of 530 nm. Anthocyanin compounds were identified based on mass spectrum and tandem mass spectrometry.

### RNA sequencing and assembly

We obtained 26 samples for transcriptome sequencing in two populations of *I*. *potaninii*, including 13 purple biological replicates and 13 yellow biological replicates (one flower bud for each sample). Besides, purple flower buds of *I. loczyi* (four samples) were collected as outgroups. All these samples were kept individually in liquid nitrogen and extracted RNA for each sample with Eastep® super total RNA extraction kit (Promega, CHINA). cDNA libraries were built according to Illumina’s recommendations, and then paired-end reads were generated on Illumina HiSeq2500 platform at the Wuhan Frasergen Bioinformatics Co. Ltd, China.

After removing adaptor and low-quality reads, Trinity v.2.8.3 [28] was used to perform the *de novo* assembly of *I. potaninii*, yellow-flowered *I. potaninii*, purple-flowered *I. potaninii*, *I. loczyi*, and *I. atropurpurea* as previously reported [29]. Non-redundant transcripts for each assembly were acquired following the best transcript screening strategy mentioned in the website (https://github.com/trinityrnaseq/trinity_community_codebase/wiki/Trinity-best-transcript-set). For each transcript, the longest transcript was selected as representative. BUSCO [30] was used to evaluate the integrality of transcriptome assemblies against the embryophyta database.

### Differential gene expression

Differential expression analysis between two color morphs of *I. potaninii* was performed against the reference of *I. potaninii* by bowtie2 v. 2.2.9 [31], rsem v.1.2.9 [32], and edgeR v.3.2.4 [33]. The fold change value (log_2_FC), CPM value (log_2_CPM), P value, and false discovery rate (FDR) for each transcript were measured. Differentially expressed genes were identified with the settings of logFC (fold change value) > 1 and FDR (false discovery rate) < 0.05.

### Variant calling and annotation

For each individual of two-color morphs, clean reads were mapped to the reference of *I. potaninii* and converted to sam files with BWA v.0.7.17-r1188 [34]. Sorted bam files were generated with samtools v.1.9 [35, 36] and duplicates were marked with Picard-tools v.2.18.9 (Broad institute, Cambridge, USA, http://broadinstitute.github.io/picard/). SNPs were called by GATK v.4.0.6.0 [37] and filtered with Variant Filtration function of GATK, including quality-by-depth ratio (QD) ≥ 2.0 and mapping quality (MQ) ≥ 40.0. Variant sites were further removed using bcftools and vcftools v.1.9 [38](Danecek et al., 2011), involving SNP within 5 bp around a gap, possibility out of Hardy-Weinberg equilibrium (HWE) > 0.001, and missing rates > 0.05. The impacts of variants were classified with the SNPeff [39].

### Single-copy orthologs and selection analysis

We predicted open reading frames (ORFs) of all assembled transcripts with TransDecoder against the Swissprot and Pfam databases. OrthoMCL [40] was used to identify the single-copy orthologs among purple-flowered *I. potaninii*, yellow-flowered *I. potaninii*, and two outgroups (*I. loczyi* and *I. atropurpurea*). Strict single-copy orthologs were identified among the two-color morphs and two outgroups (1: 1: 1: 1: 1). For each single-copy orthologous group, we aligned the protein-coding sequences and constructed the ML phylogenic trees with ParaAT [41] and IQ-TREE [42].

To investigate the selective pressure occurred in yellow-flowered *I. potaninii*, the codeml program included in PAML v.4.4 [43] was used to calculate the ratio of nonsynonymous to synonymous divergence (dN/dS = ω) and evaluate the fit of branch models and branch-site models. Foreground branch (yellow-flowered *I. potaninii*) and background branches (all other branches) were set for each single-copy orthologous phylogeny. Branch model analysis was performed for differentially evolving events, including null one-ratio model (The branch model M_0_, all sites in the sequence having the same ω value) and two-ratio model (foreground branch (ω_yellow_) was significantly different from that for all other branches (ω_purple_)). Branch-site model analysis was then used to test the signatures of positive selection in yellow-flowered *I. potaninii* if significantly elevated ω values occurred, including null model (The branch-site model Model A1_null) and alternative model (The branch-site model Model A_alternative). Likelihood ratio tests were used to compare the fit of models between null model and alternative model.

### KEGG and GO annotation

Gene ontology (GO) [44] (Blast2go v.5) and KEGG (KAAS, KEGG Automatic Annotation Server) [45, 46] were utilized to characterize the major biological functions and associated pathways of assembled transcripts, differentially expressed genes and positively selective genes.

### qRT-PCR validation

Five yellow-flowered samples and four purple-flowered samples were randomly selected to check the gene expression of differentially expressed genes involving in flower color variation of *I. potaninii*. qRT-PCR was conducted *for F3H* with 2.0 µg reverse-transcribed RNA as previously described [29]. All primers used for this study were listed in Table S3. The level of differential gene expression between purple and yellow flowers were determined by T-test with SPSS software (IBM statistic 20).

### Electronic supplementary material

Below is the link to the electronic supplementary material.

Supplementary Material 1

Supplementary Material 2

## Data Availability

The clean sequence data reported in this paper have been deposited in the Genome Sequence Archive in BIG Data Center, Beijing Institute of Genomics (BIG), Chinese Academy of Sciences, under accession numbers CRA007684 that are publicly accessible at http://bigd.big.ac.cn/gsa.
