# Supplementary material for: Flower color polymorphism of a wild Iris on the Qinghai-Tibet plateau
Source: BMC Plant Biol. 2023 Dec 9;23:633. doi: 10.1186/s12870-023-04642-9 (PMC10709947; doi:10.1186/s12870-023-04642-9)
Supplement: Supplementary file 2 — Supplementary Material 2 [file 12870_2023_4642_MOESM2_ESM.docx]

Supplementary Material

Flower color polymorphism of a wild *Iris* on the Qinghai-Tibet Plateau

Zhi-Li Zhou^1†^, Guang-Yan Wang^1†^, Xi-Long Wang^2^, Xiao-Juan Huang^1^, Zhang-ShiChang Zhu^1^, Lin-Lin Wang^1^, Yong-Ping Yang^1*^, Yuan-Wen Duan^1*^

^1^ Germplasm Bank of Wild Species, Yunnan Key Laboratory of Crop Wild Relatives Omics, Institute of Tibetan Plateau Research at Kunming, Kunming Institute of Botany, Chinese Academy of Sciences, Kunming, Yunnan 650201, China, Yunnan 650201, China.

^2^ Tibet Plateau Institute of Biology, Lhasa, Xizang 850001, China.

†The two authors contributed equally.

*** Correspondence:** Yuan-Wen Duan (duanyw@mail.kib.ac.cn), and Yong-Ping Yang (yangyp@mail.kib.ac.cn). Address: Lanhei Road 132, Heilongtan, Kunming, Yunnan, P. R. China. Phone number: 86 871 65223231

There are seven Supplementary figures.

## Supplementary Figures


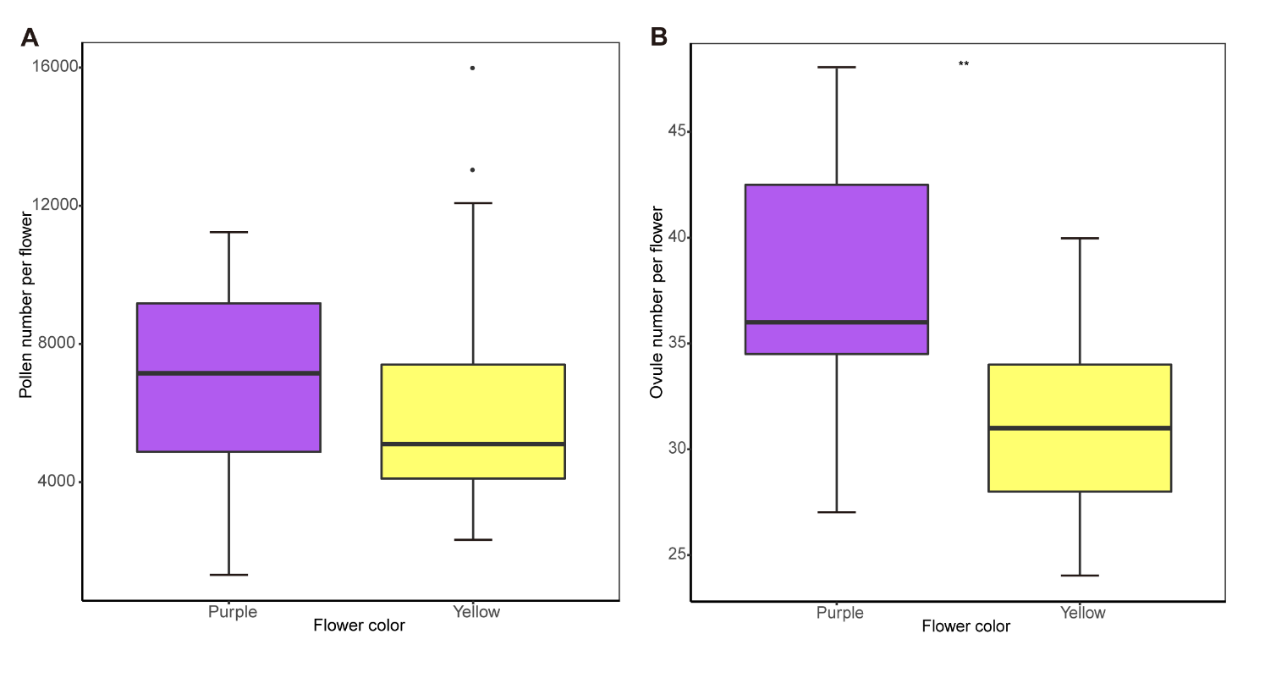


**Figure S1. Differences of pollen and ovule number per flower between purple-flowered and yellow- flowered *I. potaninii*.** Pollen number per flower is similar in both yellow and purple flowers (A), but purple flowers significantly produce more ovules than yellow flowers (B, T-test, P=0.01, asterisk indicates the 0.01 level).


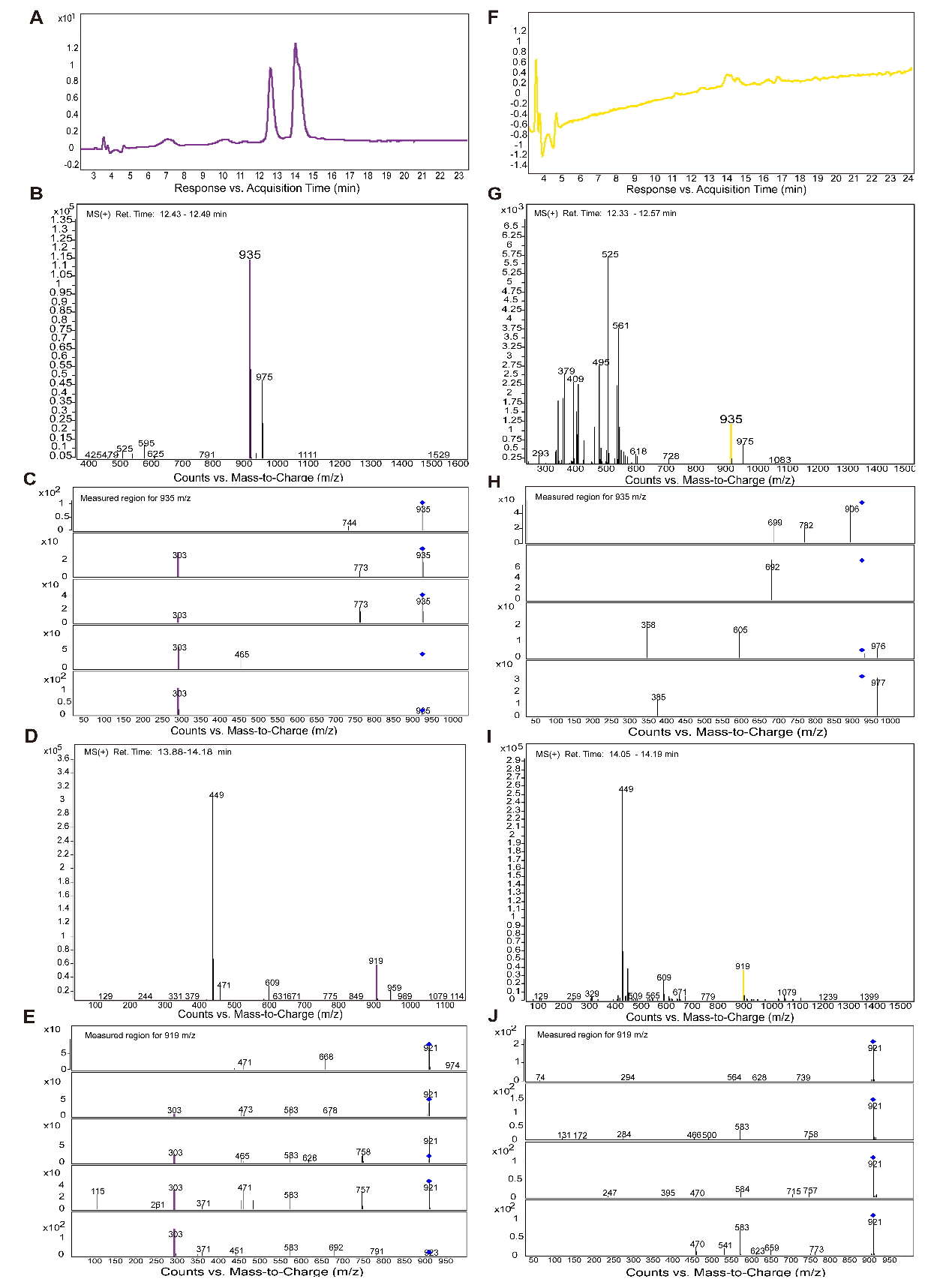


**Figure S2. liquid chromatography tandem mass spectrometry (****LC-ESI-MS/MS) results of anthocyanins in purple-flowered (A, B, C, D, E) and yellow-flowered *I. potaninii* (F, G, H, I, J).** Mass spectra were recorded between 100 m/z and 1500 m/z. According to the main detected peak, 935 m/z and 919 m/z were selected and then fragmented in MS^2^ analyses. Delphinidin derivatives (m/z 935 and m/z 919) were the main compounds in purple flowers whereas no delphinidin derivatives was identified as the dominant anthocyanins in yellow flowers.

**
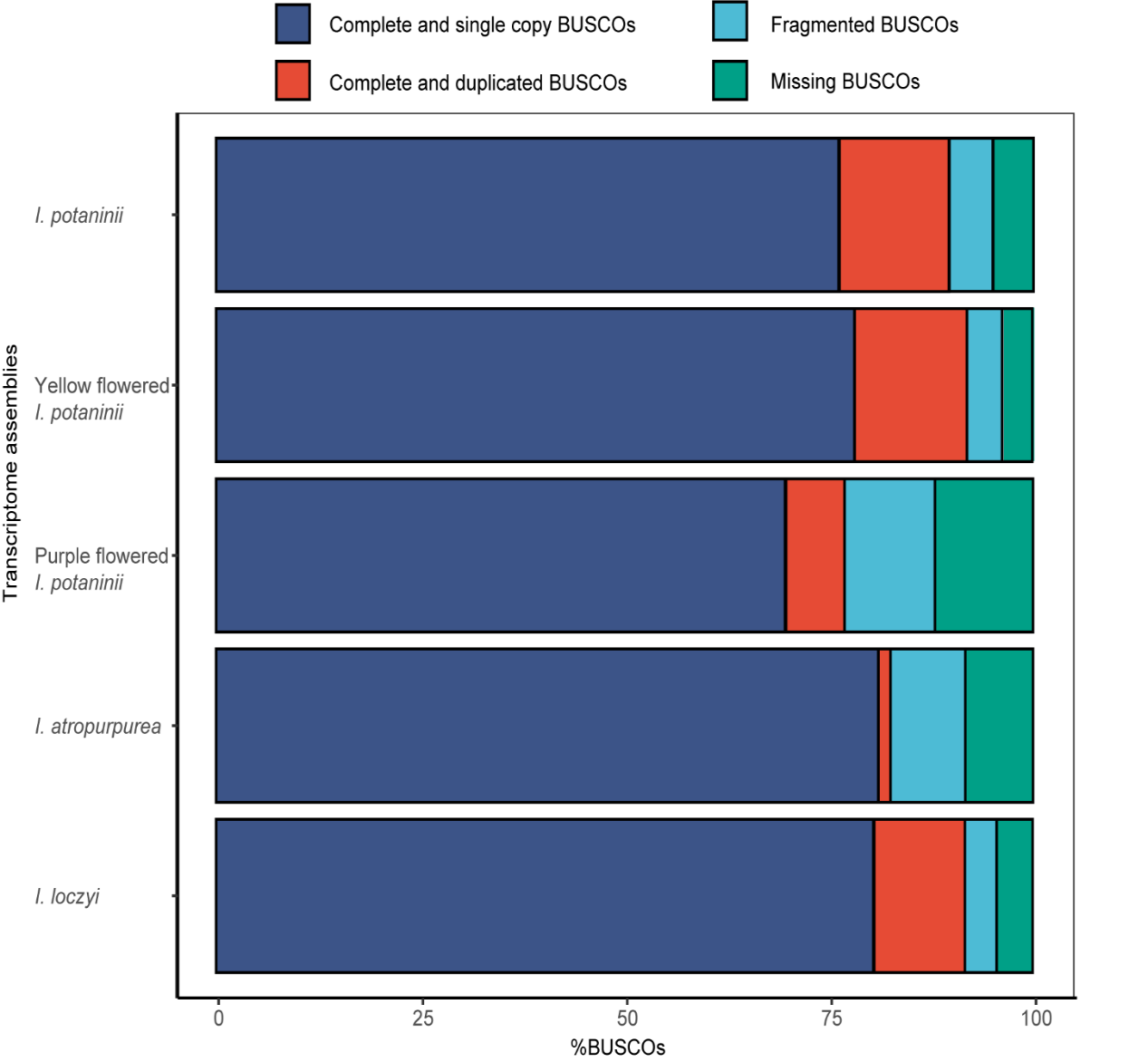
**

**Figure S3. BUSCO assessments of five transcriptome assemblies.** The high proportion of complete BUSCOs of assemblies indicate that these assemblies are of credible quality.

**
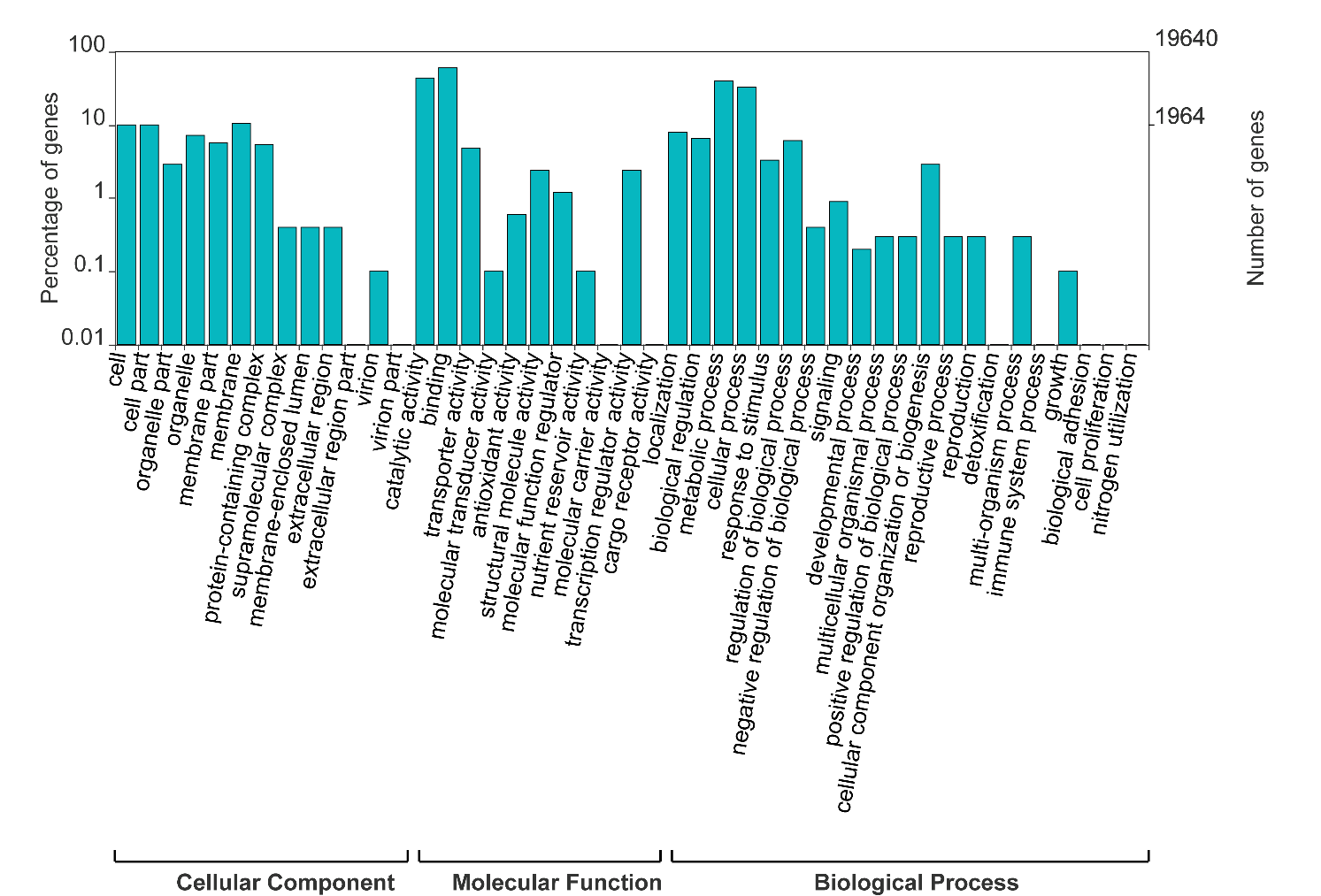
**

**Figure S4. GO annotation of transcriptome assembly of *I. potaninii* from all color-morph individuals of *I. potaninii*.** Altogether 19,640 transcripts are assigned to the GO database, including 17,810 transcripts annotated with molecular function, 10,308 transcripts annotated with biological process, and 3,818 transcripts annotated with cellular component.


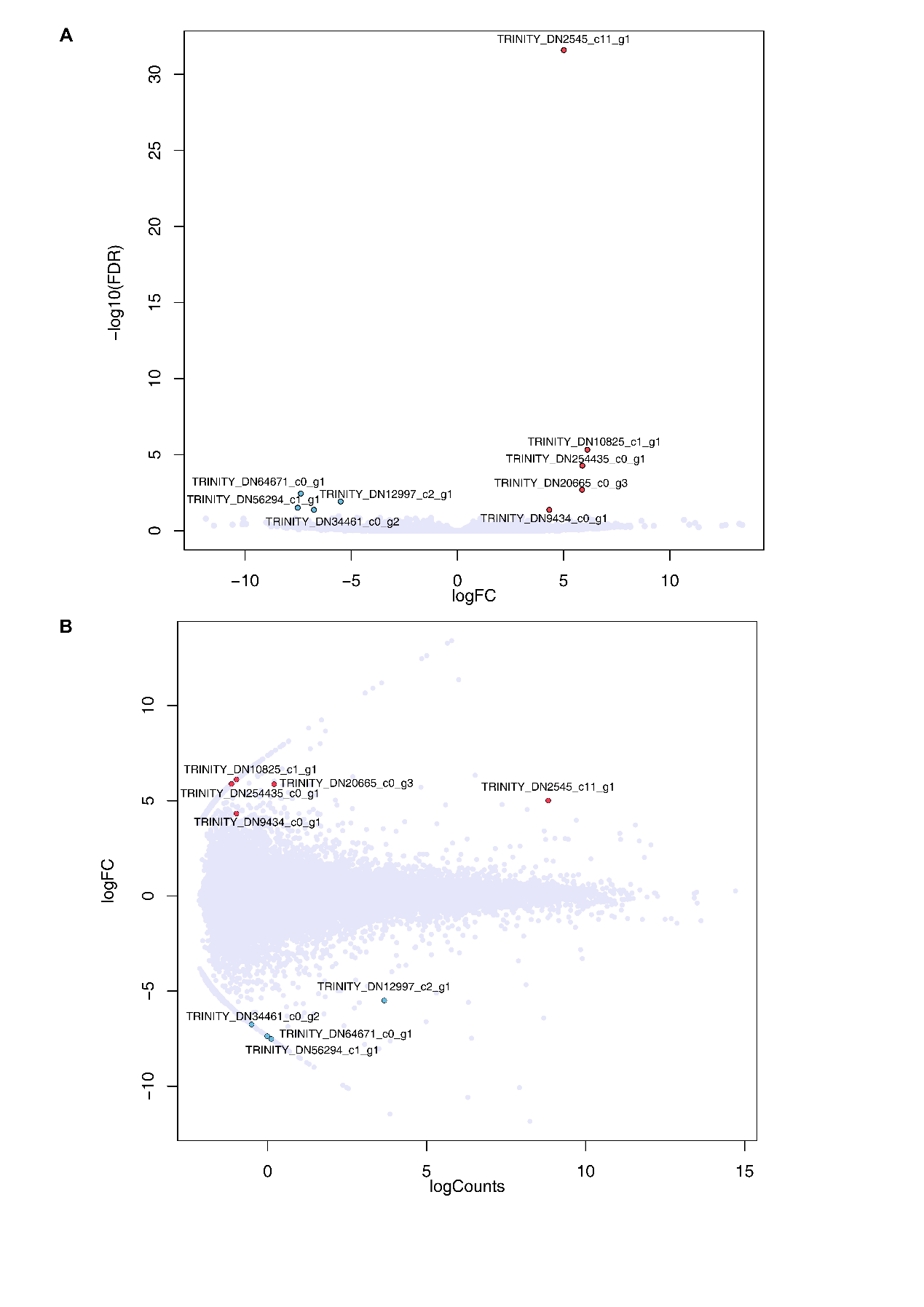


**Figure S5. volcano plot (A) and MA plot (B) of differential expression in *I. potaninii*.** Altogether nine genes (annotated with gene name) showed differential expression (logFC=1, P < 0.05) where red points indicated up-regulation in purple flowers and blue points indicated down-regulation in purple flowers compared with yellow flowers.

**
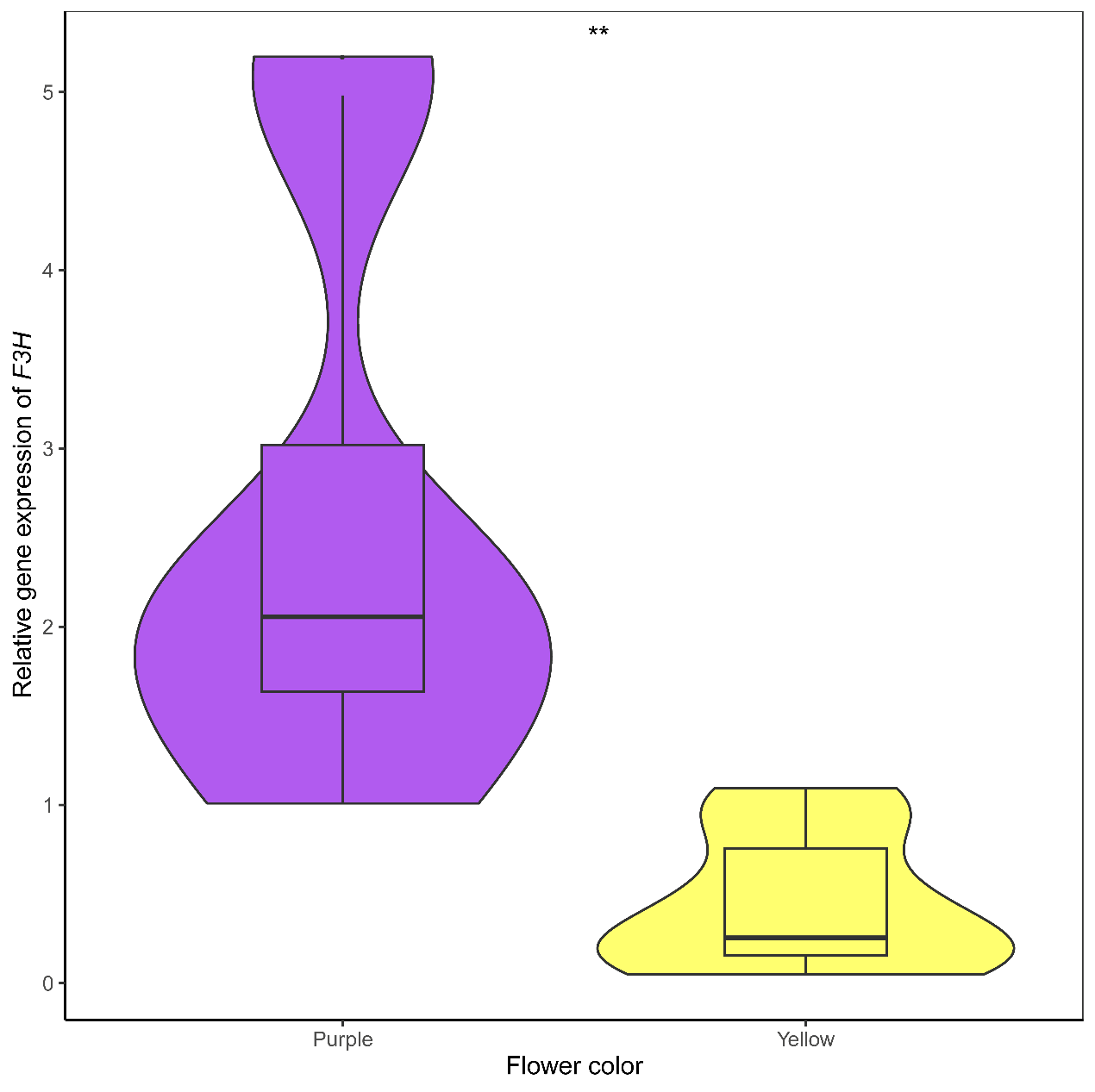
Figure S6. The mRNA expression levels of the *F3H* detected by qRT-PCR** (T-test, P = 0.007, asterisk indicates the 0.01 level).

**
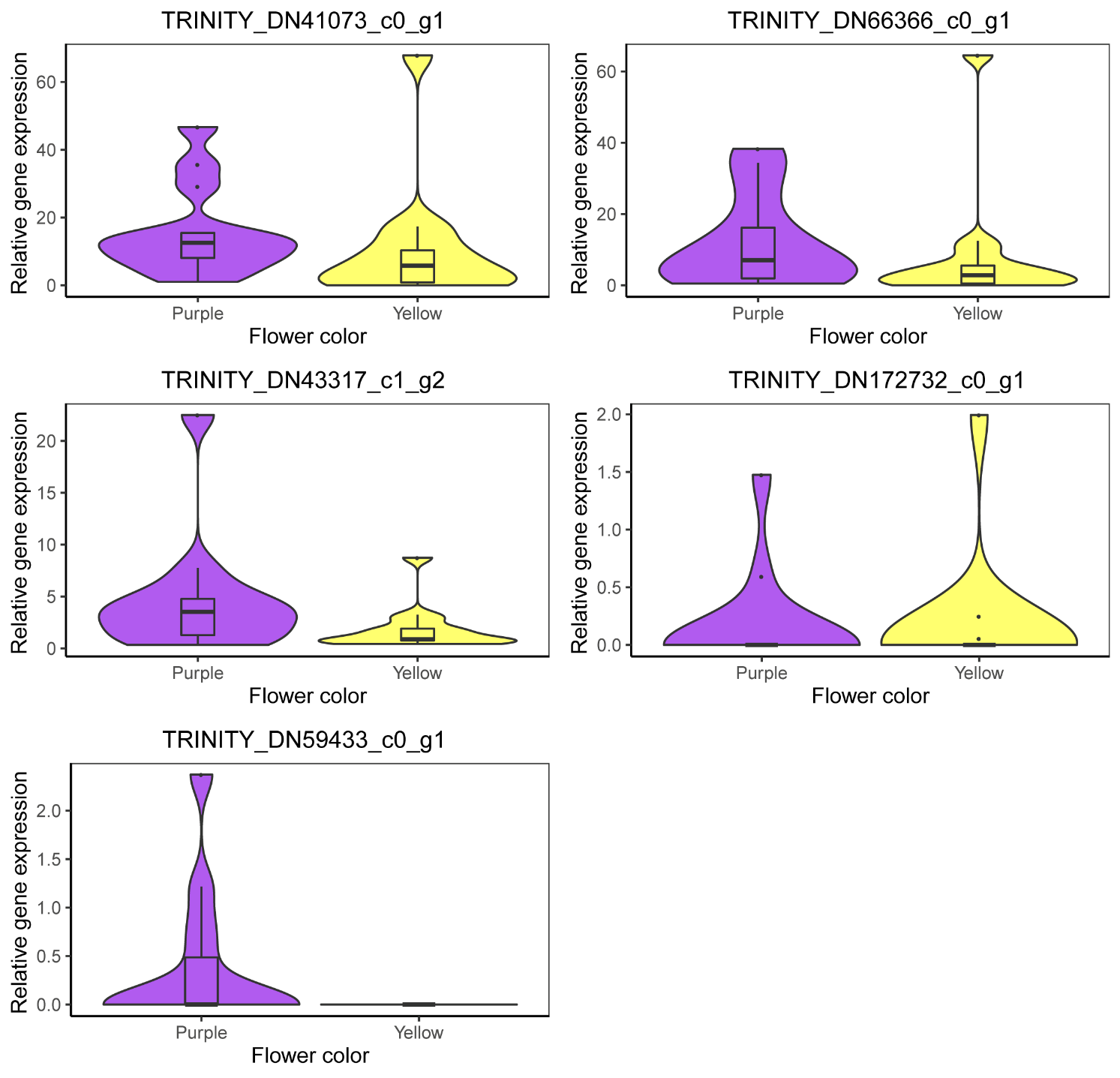
**

**Figure S7. Gene expression of *F3H* gene copies in *I. potaninii*.** Of the ten *F3H* genes, except one *F3H* showed significantly higher expressional level in purple-flowered plants than yellow-flowered ones, nine gene copies of *F3H* showed stable expression in both yellow-flowered and purple-flowered samples (Due to the very low expression of four gene copies, we only exhibit five gene copies here).
